# Stability of Strong Species Interactions Resist the Synergistic Effects of Local and Global Pollution in Kelp Forests

**DOI:** 10.1371/journal.pone.0033841

**Published:** 2012-03-16

**Authors:** Laura J. Falkenberg, Bayden D. Russell, Sean D. Connell

**Affiliations:** Southern Seas Ecology Laboratories, School of Earth and Environmental Sciences, University of Adelaide, South Australia, Australia; National Institute of Water & Atmospheric Research, New Zealand

## Abstract

Foundation species, such as kelp, exert disproportionately strong community effects and persist, in part, by dominating taxa that inhibit their regeneration. Human activities which benefit their competitors, however, may reduce stability of communities, increasing the probability of phase-shifts. We tested whether a foundation species (kelp) would continue to inhibit a key competitor (turf-forming algae) under moderately increased local (nutrient) and near-future forecasted global pollution (CO_2_). Our results reveal that in the absence of kelp, local and global pollutants combined to cause the greatest cover and mass of turfs, a synergistic response whereby turfs increased more than would be predicted by adding the independent effects of treatments (kelp absence, elevated nutrients, forecasted CO_2_). The positive effects of nutrient and CO_2_ enrichment on turfs were, however, inhibited by the presence of kelp, indicating the competitive effect of kelp was stronger than synergistic effects of moderate enrichment of local and global pollutants. Quantification of physicochemical parameters within experimental mesocosms suggests turf inhibition was likely due to an effect of kelp on physical (i.e. shading) rather than chemical conditions. Such results indicate that while forecasted climates may increase the probability of phase-shifts, maintenance of intact populations of foundation species could enable the continued strength of interactions and persistence of communities.

## Introduction

A few strong interactions often contribute disproportionately to maintaining the composition and function of an ecosystem by modifying both the physical conditions and species interactions within [1], [2], [3]. Key species can maintain ecosystem composition not only by forming biological habitats whose physical environment facilitates their own recruitment, but also by dominating competitors that would otherwise inhibit this process. Such organisms, variously called ‘foundation species’ or ‘ecosystem engineers’, create stable conditions for other dependent species [3], [4]. The inhibition of competitors associated with contrasting physical conditions and species interactions, therefore, enhances the stability of systems centered on these foundation species [5].

As human activities continue to modify abiotic conditions, there is increasing concern that such strong interactions will be altered (e.g. the sea *Pisaster ochraceus* may be less effective at consuming mussels [6]). Reduction in the strength of interactions could disrupt the persistence of entire biological communities, ranging from kelp forests to seagrasses and coral reefs in the marine realm, and grasslands to forested ecosystems in the terrestrial realm. In the marine realm, the coastal zone is an area in which high productivity and species diversity coincide with human activity and this area is set to be further influenced by the effects of a changing climate [7]. Altered land use and ensuing discharges to the marine environment elevate nutrient concentrations at local scales, with the extent of change ranging from strong enrichment in urban areas to little or no change in agricultural and natural systems [8], [9], [10]. These waters will also absorb approximately 30 percent of the atmospheric CO_2_ produced by human populations globally, leading to gradual ocean acidification [11], [12]. While there is recent recognition that these alterations of the physical environment will affect species interactions [13], [14], [15], [16] experiments to date have not progressed sufficiently to identify how they will affect biological communities dominated by foundation species such as kelp.

Australian kelp are habitat-forming species whose persistence has been enabled by their self-facilitation of recruitment through the competitive exclusion of opportunistic turf-forming algae [17]. When kelp canopies are lost, turfs rapidly colonise space and their sediment-trapping morphology inhibits the recruitment of juvenile kelp and re-formation of kelp forests [18], [19]. Under conditions of severely elevated nutrients, these naturally-ephemeral turfs persist in fragmented canopies [10], [20] to cause intergenerational decline and collapse of the kelp community [8]. Turfs, therefore, mediate the effect of nutrient-driven loss of kelp forests and often constitute a vital component in the indirect effects of pollution on habitat loss.

Under moderate scenarios of nutrient pollution, it is possible that kelp forests can persist by continuing to exclude turfs [10]. Similarly, the elevation of CO_2_ over the near-future may not alter the strength by which kelp suppress turfs. While susceptible to many other human-altered conditions, kelp meiospores are anticipated to germinate successfully under near-term enrichment of CO_2_ conditions [21]. Furthermore, productivity of ensuing recruits and subsequent individuals may be increased under elevated CO_2_ [22]. Evidence to date, however, suggests moderate increases of CO_2_ facilitate greater covers and biomass of turf, potentially turning them from ephemeral to persistent habitats [16], [23]. It remains unknown whether the competitive dominance of kelp over turf, (i.e. an interaction of particular concern to the regeneration of kelp) is likely to be reduced or increased under the combined influence of moderate nutrient and CO_2_ pollution. We consider the model that elevated CO_2_ may assist kelp sustainability despite the greater potential for turfs to persist.

We tested the hypothesis that a foundation species would continue to suppress its key competitor under conditions of moderate forecasted levels of pollution which have the potential to favour its competitor's expansion. That is, we assessed if the competitive dominance of kelp over turfs [17] would continue under moderate forecasted levels of local (i.e. nutrient) and near-term global pollution (i.e. CO_2_) and their known synergy [16].

If the strength of interactions involving foundation species are maintained despite the increasingly novel conditions brought about by human activities, then phase-shifts may be avoided. Such phase-shifts are not uncommon, but anticipating them has been problematic because many involve indirect effects [24] for which the impact of one species (e.g. kelp) on another (e.g. turf) requires knowledge of a third element that is inadequately understood (e.g. synergies among pollutants). Our study addresses a reasonably widespread challenge of forecasting the ecology of phase-shifts under future climates.

## Materials and Methods

### Experimental design

The effects of kelp removal (*Ecklonia radiata*), increased CO_2_ and elevated nutrients were tested on the turf-forming algae in a mesocosm experiment conducted in an open boat harbour located within Gulf of St. Vincent at Outer Harbour, Adelaide, South Australia (34.473395°S, 138.292184°E) (detail in “Experimental mesocosms” below). The effects of treatments on mesocosm water column physicochemical parameters were also quantified. Experimental mesocosms had combinations of kelp (present *v.* absent), CO_2_ (current *v.* future) and nutrients (ambient *v.* elevated) in a crossed design. Three replicate mesocosms were used per treatment combination, with replicate specimens of algal turfs in each mesocosm (*n* = 5). Treatments were maintained for 90 days between August and November 2009. Kelp were either present at densities similar to those observed at the collection site (9–11 m^−2^, or 3 kelp per mesocosm) or absent, as is observed on many developed coastlines, including Adelaide [10], [20]. Target [CO_2_] were based on the current ambient (current; 280–380 ppm) and the IS92a model scenario for atmospheric CO_2_ concentrations in the year 2050 (future; 550–650 ppm), which is derived from model predictions by Meehl et al. [25] (Table S4). The elevated nutrient treatment was designed to result in concentrations similar to those moderate enrichments experienced in waters off the coast of metropolitan Adelaide [10].

### Turf-forming algae

The specimens of turf-forming algae used in the experiments were collected from rocky reef with areas of turfs adjacent to kelp canopies at Horseshoe Reef, Gulf of St. Vincent, South Australia (35.13757°S, 138.46266°E). Turfs (mainly *Feldmannia* spp.) were collected from outside the kelp canopy still attached to their natural substratum (approximately the same size, 5×5 cm) and placed in holding mesocosms for eight weeks before the experiment commenced to enable acclimation to conditions in the mesocosms. Following this acclimation period five specimens of turf-forming algae were randomly assigned to each experimental mesocosm in which conditions were gradually altered over a further two week period until they reached the pre-designated experimental levels. Turf response to treatments was quantified in terms of change in percentage cover, final percentage cover and dry mass per standard area. To quantify the percentage cover of turf on each experimental specimen, a 2.5×2.5 cm quadrat was placed over the specimen within which the percentage cover was visually estimated to the nearest 5 percent. This measurement was made at the beginning (day 0; mean ± s.e. across all samples, 28.83±1.97%; three-way ANOVA detected no significant difference among samples placed in the different treatments, all *p*>0.05) and end (day 64) of the experimental period (see [26]). Change in percentage cover was then calculated by subtracting the initial percent cover from the final percent cover, while final percentage cover was that measured on day 64. Dry mass of algae was measured at the completion of the experiment (day 90) from a standard area of each specimen (2.5×2.5 cm). All algae was carefully scaped from the specimen using a razor into a pre-weighed aluminium tray, rinsed with fresh water to remove excess salt and dried to a constant weight at 60°C for 48 h before weighing (see [16], [23]).

### Experimental treatments: kelp, CO_2_ and nutrient addition

Kelp used in the experiments were collected from rocky reef adjacent to the location from which turfs were collected. Individual kelp of approximately the same size (length from bottom of stipe to tip of central lamina, mean ± SE; 32.81±1.92 cm) were collected still attached to their natural substrate and acclimated in holding mesocosms for eight weeks before the experiment commenced. Three individual kelp were then placed in each of the appropriate treatment mesocosms. The effect of kelp on light in the tanks was quantified by taking measurements using an underwater radiation sensor (Li-Cor LI-250, Nebraska, USA).

Experimental [CO_2_] of seawater in mesocosms was maintained by directly diffusing CO_2_ gas into mesocosms when required and was controlled using temperature compensated pH probes and automatic solenoid controllers (Sera, Heinsberg, Germany). Calibration of probes was checked on a daily basis and, if necessary, recalibrated using NBS calibration buffers to 0.01 pH units. The pH of mesocosms exposed to the elevated CO_2_ treatment was gradually reduced from ambient (8.15) to the experimental level (target: 7.95; measured: 7.91–7.95, see Table S4 for detail) over a two-week period (approximately 0.01 pH units per day). Total Alkalinity (TA) of seawater in mesocosms was measured weekly using colorimetric titration (Hanna Instruments, Woonsocket, RI, USA). Concentrations of *p*CO_2_ and bicarbonate (HCO_3_^−^) were then calculated from measured TA, pH, salinity and temperature using the CO2SYS program for Excel [27] with constants from Mehrbach et al. [28], as adjusted by Dickson and Millero [29].

Nutrients were enhanced using Osmocote Plus® (Scotts, Australia) controlled release fertiliser which releases a combination of nutrients at a set rate over the life of the pellet (6 month release: 15, 5, 10 N-P-K), with the nutrient concentration released proportional to weight of the fertiliser [30]. Osmocote has successfully been used in previous studies of this system to manipulate nutrient concentrations (e.g. [16], [31]). Osmocote pellets were placed in a nylon mesh bag (1 mm mesh size) and attached to the bottom of each appropriate mesocosm (10 g per mesocosm). The concentration of the supplied nutrients was quantified by regularly collecting water samples using 25 mL sterile syringes, which were filtered (0.45 µm glass fibre) and immediately frozen. Samples were later analysed on a Lachat Quickchem 8500 Flow Injection Analyser (Hach, CO, USA) for ammonia, phosphate and NO_X_ (nitrite+nitrate). Additionally, to quantify the effect of elevated nutrients in the absence of biota, a trial was conducted whereby 10 mesocosms identical to the field mesocosms were established in the laboratory and maintained for five weeks between March and April 2011. Using the same methods as in the field, 10 g of Osmocote was added to half of these tanks, with water samples being regularly analysed from all mesocosms.

### Experimental mesocosms

The closed, experimental mesocosms were moored in a boat harbour adjacent to the Gulf of St. Vincent at Outer Harbour, Adelaide, South Australia. The boat harbour is protected from the predominant swell by a breakwall, but which has a channel wide enough to allow high flushing rates. The mesocosms were moored alongside a system of floating pontoons that move up and down with the tides, and held in place by an array of vertical pilings. Mesocosms (L×W×H: 0.5×0.5×1 m) were filled with natural seawater pumped directly from the harbour, therefore, the initial seawater chemistry (i.e. before experimental manipulation) was characteristic of these waters. While this water is not different from that adjacent to the harbour and is representative of the oligotrophic coastlines of South Australia, the quality of water used in the mesocosm experiments may not have been ambient relative to the collection site. During the experimental period one-third of the seawater was removed from each mesocosm and replaced with fresh seawater weekly to maintain water quality. The mesocosms were located in full sunlight and consequently experienced diurnal and seasonal fluctuations in sunlight and temperature.

### Analyses

Three-factor Analysis of Variance (ANOVA) was used to test the response of algal turfs to experimental conditions (change in percentage cover, final percentage cover and dry mass per area of turfs). The three factors of kelp, CO_2_ and nutrients were treated as fixed and orthogonal, with two levels in each factor (Kelp: present *v.* absent; CO_2_: current *v.* future; Nutrient: ambient *v.* elevated). Data for the five algal specimens within each mesocosm were averaged and analysed with mesocosms as replicates (*n* = 3). Three-factor ANOVA (as described above) was used to test the water column physicochemical parameters of mesocosms with measurements averaged across days (pH, TA, *p*CO_2_, HCO_3_^−^ and temperature *n* = 8 days; light *n* = 1 day; ammonia, phosphate and NO_X_ in field *n* = 6 days; ammonia phosphate and NO_X_ in laboratory *n* = 20 days) and mesocosms used as replicates (*n* = 3 for field; *n* = 5 for laboratory). Where significant treatment effects were detected, Student–Newman–Keuls (SNK) *post hoc* comparison of means was used to determine which factors differed. The magnitude of effects (ω^2^) was calculated [32], [33] to assess which factor, or combination of factors, primarily contributed to the response of turfs (in terms of change in percentage cover, final percentage cover and dry mass per area) under experimental treatments.

## Results

### Turf-forming algae

**Figure 1:**
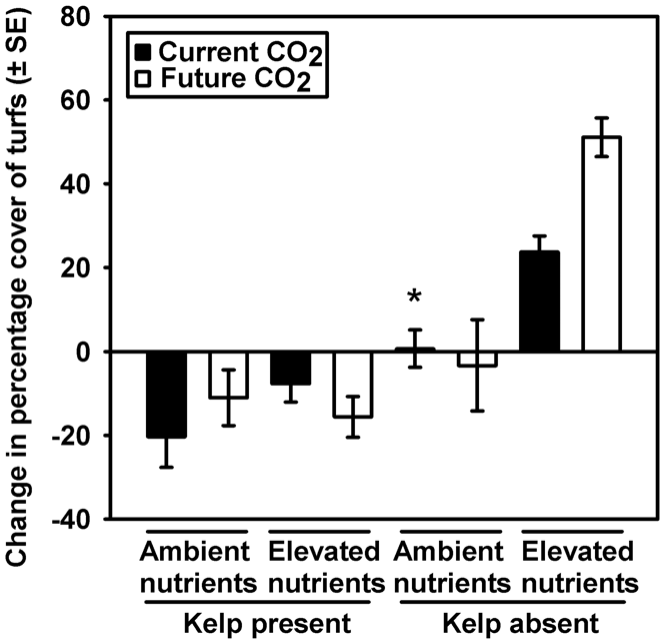
The change in percentage cover of turf-forming algae (final – initial measurement) that were transplanted from ambient conditions* to different combinations of Kelp (present *v.* absent), Nutrients (ambient *v.* elevated) and CO_2_ (current *v.* future). * Ambient conditions = turfs growing in canopy gaps under ambient concentrations of nutrients and CO_2_.

A key result was that the negative response of turfs to canopies was of similar magnitude across all treatments of pollution and their combinations. There was no change in the percentage cover of turfs under ‘ambient conditions’ (i.e. the experimental treatments of ambient nutrients and current CO_2_ and no kelp canopy) from the beginning to end of the experimental period (Figure 1). The treatment of largest influence was the presence or absence of kelp canopies (ω^2^ = 0.53; Table S1). In the absence of kelp, elevated nutrients and CO_2_ positively affected percentage cover in a multiplicative rather than additive manner (Figure 1; Table S1; SNK test of Kelp×Nutrient×CO_2_ interaction). In the presence of kelp, the percentage cover of turfs was reduced below that of ‘ambient conditions’, with neither elevated CO_2_ or nutrients having a significant effect, either in isolation or combination (Figure 1; Table S1; SNK test of Kelp×Nutrient×CO_2_ interaction).

**Figure 2:**
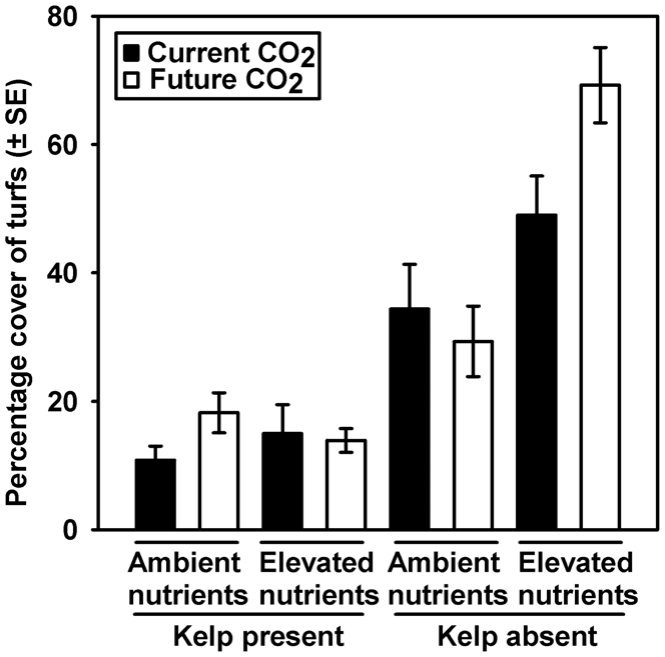
The final percentage cover of turf-forming algae that were transplanted from ambient conditions (as defined in **Figure 1**) to different combinations of Kelp (present *v.* absent), Nutrients (ambient *v.* elevated) and CO_2_ (current *v.* future).

A synergistic interaction occurred between the simultaneous effects of kelp loss and multiple pollutants (i.e. CO_2_ and nutrients), with these treatments resulting in percentage covers (Figure 2; mean ± SE; 69.25±5.88%) which cannot be predicted from the independent effects of kelp in the absence of elevated CO_2_ and nutrients (i.e. kelp absent – present = 23.50%), future CO_2_ in the absence of kelp and elevated nutrients (i.e. future CO_2_ – ambient CO_2_ = −5.00%) and elevated nutrients in the absence of kelp and elevated CO_2_ (i.e. elevated nutrients – ambient nutrients = 14.67%). Elevated CO_2_ alone had no detectable effect in the absence of kelp, but caused greater covers of turfs when combined with elevated nutrients (Figure 2; Table S2; SNK test of Kelp×Nutrient×CO_2_ interaction). The treatment of largest influence was the presence or absence of kelp canopies (ω^2^ = 0.78; Table S2). Canopies of kelp restricted the cover of turf to an average of 19.84% less than ‘ambient conditions’, and 54.76% less than the combination of elevated CO_2_ and nutrients (Figure 2), demonstrating the strong competitive effects of kelp over turfs under both ambient and forecasted conditions. Importantly, this competitive effect was consistent across the treatments of elevated CO_2_ and nutrients both when they were manipulated in isolation and combination (Figure 2; Table S2; SNK tests).

**Figure 3:**
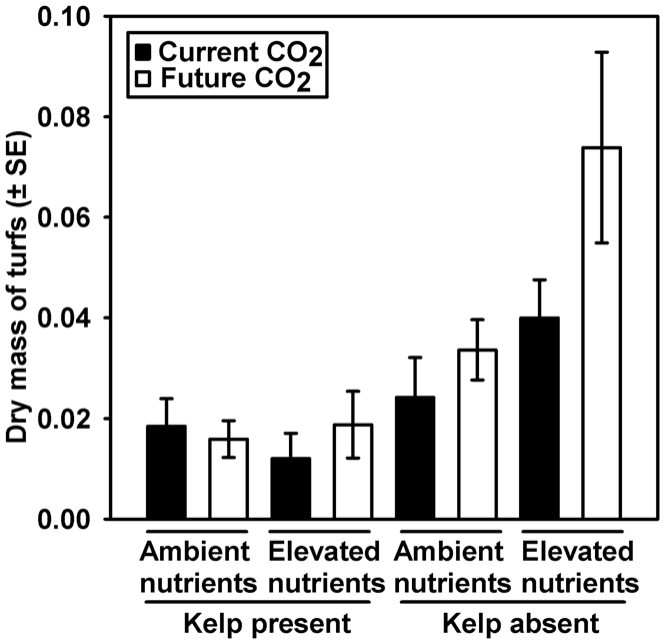
The dry mass of turf-forming algae on natural rock substrate that were transplanted from ambient conditions (as defined in **Figure 1**) to different combinations of Kelp (present *v.* absent), Nutrients (ambient *v.* elevated) and CO_2_ (current *v.* future).

The removal of kelp and elevation of CO_2_ and nutrients had positive effects on the dry mass of turf, with the greatest mass (0.07±0.02 g) when they were manipulated in combination (Figure 3; Table S3). While the presence or absence of kelp was the treatment of largest influence (ω^2^ = 0.54; Table S3), nutrients and the kelp×nutrient term also contributed strongly (ω^2^ = 0.15 for both; Table S3). Kelp and nutrients interacted such that the mass of turf was greater under elevated than ambient nutrient conditions, with this effect restricted in the presence of kelp (Table S3; SNK of significant Kelp×Nutrient interaction).

### Water column physicochemical parameters

The concentration of ammonia, phosphate and NO_X_ (nitrate+nitrite) quantified in the field mesocosms was significantly higher in elevated (mean ± SE; ammonia 0.0406±0.0025 mg L^−1^, phosphate 0.0091±0.0002 mg L^−1^, NO_X_ 0.0060±0.0002 mg L^−1^) than ambient nutrient treatments (ammonia 0.0296±0.0021 mg L^−1^, phosphate 0.0079±0.0001 mg L^−1^, NO_X_ 0.0054±0.0002 mg L^−1^) (Table S4, S5; Figure S1a, c, e). These relatively small differences (e.g. NO_X_<0.0001 mg L^−1^) indicate the elevated nutrients were being used by the algae. This interpretation is supported by the additional laboratory-based mesocosm trials, testing the effects of nutrient enrichment in the absence of algae. That is, the measurable concentrations of nutrients in the elevated nutrient treatments were substantially greater (ammonia 0.2652±0.0320 mg L^−1^, phosphate 0.1285±0.0068 mg L^−1^, NO_X_ 0.3796±0.0255 mg L^−1^) than ambient nutrient treatments (ammonia 0.0346±0.0053 mg L^−1^, phosphate 0.0272±0.0033 mg L^−1^, NO_X_ 0.1222±0.0050 mg L^−1^) (Table S4, S5; Figure S1b, d, f).

TA, *p*CO_2_, and HCO_3_^−^ were increased in treatments where CO_2_ was experimentally elevated (Table S4, S5; Figure S2b, c, d), while pH was reduced under future CO_2_ compared with current CO_2_ conditions (Table S4, S5; Figure S2a). Light was reduced where kelp were present (70.34±11.15 µmol m^−2^ s^−1^) compared to where they were absent (1316.44±59.57 µmol m^−2^ s^−1^) (Table S4, S5). Temperature was not significantly different among treatments (Table S4, S5).

## Discussion

Over 30 years ago, Harrison [34] suggested that there was a need to understand not only the behavior of a community under ‘normal or good conditions’, but also its response to unusual or stressful conditions. Since then, research considering the effects of stressful conditions created by human activities has often focused on identifying the community response to highly-modified conditions (e.g. [35], [36]). A more pressing contemporary concern, however, is whether moderate near-term alterations will be of a sufficient magnitude to drive changes in community interactions. Potential exists that near-term future conditions may reduce the capacity of foundation species to suppress competitors whose expansion would otherwise cause communities to shift to, and be maintained in, a contrasting state (e.g. [19]). Whilst severe pollution, such as nutrient conditions associated with urban coasts [10], is known to reduce the capacity of kelp forests to recover from disturbance (i.e. resilience) [19], intact kelp forests may be quite stable in the face of similar sets of stressors, of a lesser magnitude, such as coasts associated with agriculture [10]. Although near-term forecasted environmental conditions are anticipated to facilitate competitors and increase the probability of loss of foundation species (e.g. the strong positive synergistic effect of increasing nutrient and CO_2_ concentrations on turf [16]), the current study suggests that where kelp canopies are retained their mere presence may be sufficient to continue to suppress a key competitor (e.g. turfs), despite the synergistic effects of moderate elevation of local (i.e. elevated nutrients) and global pollutants (i.e. forecasted CO_2_). As the conditions that promote community resistance may be different from those that favour resilience, recognizing the factors that affect persistence rather than recovery could assist in forecasting their effects on these normally robust and diverse natural systems [37].

The synergistic responses of kelp competitors to multiple pollutants (i.e. turf response to CO_2_×nutrients ([16], this study), supports the model that multiple stressors can combine to produce conditions which increase the likelihood of phase-shifts [38]. Consequently, researchers have been increasing their focus to identify those sets of stressors which combine to produce effects that cannot be anticipated by adding their isolated effects [39]. The frequency and magnitude of non-additive responses are surprisingly common, to the extent that our concept of resource limitation has shifted from an earlier paradigm of single-resource limitation [40] towards that of co-limitation by multiple resources [41], [42]. While ‘limitation’ can be experimentally recognised by changing the rate of processes through addition or reduction of the single relevant factor, ‘co-limitation’ is recognised as the greater response to simultaneous enrichment of multiple factors than would be expected from the sum of their individual responses [42]. The repeated observation of an interaction between CO_2_ and nutrients ([16], this study) indicates nutrients are not available in great excess relative to CO_2_, as a modest addition of CO_2_ quickly produces a limitation on nutrients. It also appears CO_2_ is not in great excess relative to nutrients, as an addition of nutrients quickly provokes a limitation on CO_2_. When CO_2_ and nutrients are added together, CO_2_ and nutrient limitation may alternate in numerous small incremental steps, ultimately producing a synergistic effect. This model may account for the observed synergy between CO_2_ and nutrients in a similar way Davidson and Howarth [43] account for the prevalence of nitrogen and phosphorous interactions [44]. Whilst this synergy would appear relevant for canopy-gaps or locations experiencing canopy loss, it is less likely to be relevant in disrupting the persistence of intact kelp forests

The mechanisms that allow kelp to suppress their competitors under conditions that would otherwise facilitate their spread may be useful to understand. Quantification of physiochemical conditions within the experimental mesocosms indicates that the mechanism driving kelp inhibition is alteration of the physical (i.e. shading) rather than chemical (i.e. nutrient or carbonate) conditions experienced by understorey species. The presence of kelp did not appear to modify either the nutrient status (i.e. ammonia, phosphate, NO_X_) or carbonate chemistry of water within the mesocosms (i.e. pH, TA, *p*CO_2_, HCO_3_^−^; see also Figure S3 for diurnal pH variation). We suspect, however, that the accelerated growth of turf in the absence of kelp is likely to obscure this potential effect by utilising the relatively moderately elevated nutrients. On biomass basis, turfs are naturally more productive (i.e. 44–77%) than surrounding canopy-forming algae in this system [45]. We consider that shading by kelp canopies provides a more powerful explanation of the suppression of turfs. This explanation is derived from classical experiments showing the effects of canopy-shade on understorey communities [46] and covers of turfs [17], [47]. Where perennial canopy species are removed, algae adapted to high light conditions, such as turfs, are then able to utilise the increased light to expand their covers [46], [48]. In contemporary algal assemblages the presence of intact kelp canopies reduces light reaching the substratum to a similar extent as that which was observed in our experimental mesocosms (i.e. a ∼95% reduction) [17], [47].

The retention of populations of foundation species seems critical in ensuring maintenance of the primary mechanism that enables the continued dominance of kelp over its competitors, in this case shading. We do, however, recognise that this conclusion is based on the assumption that communities will remain intact, maintaining the strength of interactions, a particularly important assumption for assemblages whose structure is determined by a small number of interactions centred on a single foundation species [49]. The biotic factors that influence shading tend to vary, especially when the impacts of human activities, such as canopy removal, are considered [50]. While the delivery of light flecks to the understorey during canopy movement appears important in maintaining understorey productivity, when large amounts of light become available, such as when entire plants are removed from the substratum and a gap in the canopy is produced, the influence of the canopy may be reduced and persistence of ecosystems disrupted [51]. For example, as kelp canopies are thinned, reduced in size or fragmented, the associated environmental conditions (including light) become more similar to those experienced outside the canopy [52]. Under these conditions, turfs can expand to dominate space in assemblages and inhibit the recruitment of kelp [19], [23], leading to phase-shifts over multiple generations [53].

Key species can maintain ecosystem composition through strong interactions that are often self-stabilising because they create conditions that facilitate the persistence of entire ecosystems [54]. Given that species interactions are often mediated by environmental conditions [55], [56], human activities which modify the abiotic environment have the potential to disrupt these interactions and alter the species composition of ecosystems [7], [15]. Where strong interactions maintain community structure by retarding the effects of environmental forcing, management of key species may assist in the retention of communities, even under forecasted global conditions (i.e. large-scale pollution and climate change).

In conclusion, our results show the interaction between kelp and turf may be maintained under near-term future conditions, indicating the retention of intact forests may reduce the effect of moderate pollutant enrichment in these communities. Many communities are governed by a few strong interactions (e.g. presence of kelp forests) which exert disproportionately strong community-wide effects [3]. The maintenance of intact populations of foundation species may enable these habitats to persist despite forecasted climates that would otherwise appear to increase the probability of their loss.

## Supporting Information

Table S1 **ANOVA testing the combined effect of Kelp (present** ***v.*** **absent), Nutrients (ambient** ***v.*** **elevated) and CO_2_ (current** ***v.*** **future) on the change in percentage covers of turf-forming algae.**
(TIF)

Table S2 **ANOVA testing the combined effect of Kelp (present** ***v.*** **absent), Nutrients (ambient** ***v.*** **elevated) and CO_2_ (current** ***v.*** **future) on the final percentage covers of turf-forming algae.**
(TIF)

Table S3 **ANOVA testing the combined effect of Kelp (present** ***v.*** **absent), Nutrients (ambient** ***v.*** **elevated) and CO_2_ (current** ***v.*** **future) on the final weight per area of turf-forming algae.**
(TIF)

Table S4 **Physicochemical parameters of mesocosms measured in the field (** ***n*** **= 9) and the laboratory (** ***n*** **= 3) for each treatment.** Reported are means, standard errors (S.E.), maximum and minimum values. Field ammonia, phosphate and NO_X_ were sampled weekly on six occasions, with laboratory-based mesocosms sampled on alternate days (*n* = 20 occasions). Total Alkalinity (TA), pH and temperature were simultaneously measured weekly on eight occasions, from which concentrations of *p*CO_2_ (ppm) and bicarbonate (HCO_3_^−^) (µmol kg^−1^) were calculated using constants from Mehrbach et al. [28], as adjusted by Dickson and Millero [29]. Light was measured on one occasion.
(TIF)

Table S5 **Results from ANOVA, testing the combined effect of Kelp (present** ***v.*** **absent), Nutrients (ambient** ***v.*** **elevated) and CO_2_ (current** ***v.*** **future) on the 9 physicochemical parameters measured in the field and effect of Nutrients (ambient** ***v.*** **elevated) on the 3 measured in the laboratory.** Field ammonia, phosphate and NO_X_ were sampled weekly on six occasions, with laboratory-based mesocosms sampled on alternate days (*n* = 20 occasions). Total Alkalinity (TA), pH and temperature were simultaneously measured weekly on eight occasions, from which concentrations of *p*CO_2_ (ppm) and bicarbonate (HCO_3_^−^) (µmol kg^−1^) were calculated using constants from Mehrbach et al. [28], as adjusted by Dickson and Millero [29]. Light was measured on one occasion.
(TIF)

Figure S1 **Nutrient concentrations within field (a, c, e) and laboratory (b, d, f) based mesocosms measured from beginning to end of the experiment.** Ammonia (a, b), phosphate (c, d) and NO_X_ (e, f ) under ambient nutrients (filled circles) and elevated nutrients (empty circles). Data presented are means across CO_2_ and kelp treatments. Note the different scales on the y-axes.
(TIF)

Figure S2 **Carbonate chemistry parameters in field-based experimental mesocosms measured weekly from beginning to end of the experiment.** pH (a), TA (b), *p*CO_2_ (c), HCO_3_^−^ (d) in mesocosms under current CO_2_ (filled circles) and future CO_2_ (empty circles). Total Alkalinity (TA) and pH were measured weekly on eight occasions, from which concentrations (µmol kg^−1^) of *p*CO_2_, and bicarbonate (HCO_3_^−^) were calculated. Values were calculated from measured TA and pH using constants from Mehrbach et al. [28], as adjusted by Dickson and Millero [29]. Data presented are means across different nutrient and kelp treatments.
(TIF)

Figure S3 **A representative diurnal cycle (Oct 9–10, 2009; 0630-0630) of pH for all treatment combinations.** CCO_2_, current CO_2_; FCO_2_, future CO_2_; KP, kelp present; KA, kelp absent; AN, ambient nutrients; EN, elevated nutrients.
(TIF)
